# A gene co-expression module implicating the mitochondrial electron transport chain is associated with long-term response to lithium treatment in bipolar affective disorder

**DOI:** 10.1038/s41398-018-0237-0

**Published:** 2018-09-05

**Authors:** David Stacey, K. Oliver Schubert, Scott R. Clark, Azmeraw T. Amare, Elena Milanesi, Carlo Maj, Susan G. Leckband, Tatyana Shekhtman, John R. Kelsoe, David Gurwitz, Bernhard T. Baune

**Affiliations:** 1 Discipline of Psychiatry, School of Medicine, University of Adelaide, Adelaide, SA Australia; 2 MRC/BHF Cardiovascular Epidemiology Unit, Department of Public Health and Primary Care, University of Cambridge, Cambridge, CB1 8RN UK; 3 Northern Adelaide Local Health Network, Mental Health Services, Lyell McEwin Hospital, Elizabeth Vale, SA 5112 Australia; 4 Genetics Unit, IRCCS, San Giovanni di Dio, Fatebenefratelli, Brescia, Italy; 5 Department of Cellular and Molecular Medicine, ‘Victor Babes’ National Institute of Pathology, 99-101 Splaiul Independentei, 050096 Bucharest, Romania; 6 Institute for Genomic Statistics and Bioinformatics, University Hospital Bonn, Bonn, Germany; 7 University of California San Diego and VA San Diego Healthcare System, San Diego, CA USA; 8 Department of Human Molecular Genetics and Biochemistry, Sackler Faculty of Medicine, Tel Aviv University, Tel Aviv, Israel; 9 Department of Psychiatry, Melbourne Medical School, Royal Melbourne Hospital, University of Melbourne, VIC, Australia

## Abstract

Lithium is the first-line treatment for bipolar affective disorder (BPAD) but two-thirds of patients respond only partially or not at all. The reasons for this high variability in lithium response are not well understood. Transcriptome-wide profiling, which tests the interface between genes and the environment, represents a viable means of exploring the molecular mechanisms underlying lithium response variability. Thus, in the present study we performed co-expression network analyses of whole-blood-derived RNA-seq data from *n* = 50 lithium-treated BPAD patients. Lithium response was assessed using the well-validated ALDA scale, which we used to define both a continuous and a dichotomous measure. We identified a nominally significant correlation between a co-expression module comprising 46 genes and lithium response represented as a continuous (i.e., scale ranging 0–10) phenotype (cor = −0.299, *p* = 0.035). Forty-three of these 46 genes had reduced mRNA expression levels in better lithium responders relative to poorer responders, and the central regulators of this module were all mitochondrially-encoded (*MT-ND1*, *MT-ATP6*, *MT-CYB*). Accordingly, enrichment analyses indicated that genes involved in mitochondrial functioning were heavily over-represented in this module, specifically highlighting the electron transport chain (ETC) and oxidative phosphorylation (OXPHOS) as affected processes. Disrupted ETC and OXPHOS activity have previously been implicated in the pathophysiology of BPAD. Our data adds to previous evidence suggesting that a normalisation of these processes could be central to lithium’s mode of action, and could underlie a favourable therapeutic response.

## Introduction

Bipolar affective disorder (BPAD) affects at least 2% of the population and is characterised by recurring episodes of depression, mania, or mixed affective states^1^. Mood stabilising medications are prescribed for most BPAD patients. These drugs are effective in ameliorating the symptoms of acute illness episodes^2^, as well as providing long-term protection against episode recurrence^3^. Lithium is widely considered the ‘gold standard’ mood stabiliser^3,4^ due to its uniquely protective effects against both manic and depressive episodes^5^, as well as suicide^6^. Therefore, lithium is recommended as first-line maintenance treatment for BPAD by several clinical practice guidelines^7–10^.

However, the clinical response to lithium is highly variable between individuals. While many patients achieve full symptom resolution and long-term recovery, 30% show only partial response, and a further 30% are considered poor responders^11–13^. Evidence from the literature highlights some potential clinical predictors of a favourable vs. poor response to lithium in BPAD. These include course of illness, age of BPAD onset, number of BPAD hospitalisations, and pattern of BPAD symptomatology^14,15^. Further, genetic factors are known to explain some of the clinical variability. ‘Good’ lithium responders are more likely to come from families with a higher prevalence of BPAD, and to have relatives that have also responded favourably^16–18^. Previous genetic investigations including linkage^19–21^, candidate gene^22–24^, and genome-wide association studies (GWAS)^25–27^ have implicated multiple genes as candidate mediators of the genetic component of lithium response, but the vast majority of these findings have not been replicated^28^. Most recently, a GWAS of over 2500 BPAD patients conducted by the International Consortium on Lithium Genetics (ConLi^+^Gen)^29^ has implicated an intergenic locus on chromosome 21 at genome-wide significance (*p* < 5 × 10^−08^) for association with a quantitative measure of lithium response. Long intergenic non-coding RNAs (lincRNAs) reside at this locus, suggesting they may mediate this association. However, in-depth follow-up functional characterisation is required before this genome-wide significant association can be harnessed to improve our understanding of the molecular mechanisms underlying the therapeutic effects of lithium, much less provide any clinical utility^29^.

No biological markers currently exist in psychiatric practice for the purpose of reliably stratifying subgroups of good vs. poor responders to lithium, at the outset of therapy. This has multiple implications, including the potential risk of prolonging clinical symptoms in patients that are found to respond poorly to lithium and who might otherwise have responded better to an alternative drug. Further, because long-term lithium therapy is associated with potentially serious side effects including chronic renal failure, hypothyroidism and mortality due to acute toxicity^4^, this lack of markers for personalised prescribing puts BPAD patients at additional risk.

Transcriptome-wide profiling using microarray or next generation RNA-sequencing technology constitutes a complementary approach towards identifying biological correlates of lithium response in BPAD, with the potential to elucidate the underlying mechanisms. Few such studies to date have been undertaken using patient samples but rather cultured cells that have undergone immortalisation^30–32^. Results so far have implicated, for example, apoptotic pathways^30,31^, neutral amino acid transport^30^, protein ubiquitination, and protein synthesis^32^. However, while providing valuable leads for future research, the findings of these studies have been compromised by relatively small sample sizes, associated difficulties with statistical power and multiple testing, and poor replicability^33^. Additionally, no previous gene expression study has measured gene expression correlates of long-term response to lithium treatment beyond 6 months.

In order to overcome these difficulties in the current study, we performed weighted gene co-expression network analysis (WGCNA), a hypothesis-free systems biology approach that identifies ‘modules’ of co-regulated, and therefore functionally related, genes in transcriptomic data sets. Emerging evidence indicates that such an approach can be advantageous in characterising clinical phenotypes that are highly complex, polygenic, and heterogeneous^34–36^, even when utilising transcriptomic data from relatively small sample sizes. Indeed, the recommended minimum sample size to construct a robust network using WGCNA is just 15–20 (https://labs.genetics.ucla.edu/horvath/CoexpressionNetwork/Rpackages/WGCNA/faq.html), while functional term enrichment and pathway analyses have demonstrated that co-expression networks constructed using data from small to moderate sample sizes are biologically meaningful^37,38^.

## Materials and methods

### Sample recruitment and data collection

In all, *n* = 50 BPAD patients of European ancestry (Supplementary Table 1) undergoing or who had undergone treatment with lithium were recruited from hospitals across the state of South Australia, Australia, as part of the ConLi^+^Gen consortium (Supplementary Information)^39^ and the ongoing University of Adelaide ‘Cognitive Function and Mood Study’ (CoFaMS, HREC RAH No. 111230). Written informed consent was obtained, after which lithium treatment response data were collected and a 10 mL blood sample was taken by forearm venipuncture into Vacutainer EDTA tubes. Whole-blood samples were then aliquoted into standard Eppendorf tubes; (i) 2 mL aliquots stored at −80 °C for subsequent genomic DNA (gDNA) extraction, and (ii) 1 mL aliquots stabilised with RNA*later*™ (Thermo Fisher Scientific, Norwood SA, Australia) stored at −20 °C for total RNA extraction.

### Measurement of lithium treatment response

Lithium treatment response was assessed using a previously published and validated scale called the ‘Retrospective Criteria of Long-Term Treatment Response in Research Subjects with Bipolar Disorder’ scale, also known as the ALDA scale^13,16^. The ALDA scale is described in more detail in Supplementary Information.

For analyses we directly utilised the total ALDA score (i.e., range 0–10) as a ‘continuous’ measure of lithium treatment response, and we also defined a ‘dichotomous’ variable with an ALDA score cutoff ≥ 5 to represent ‘responders’ vs. ‘non-responders’ (Supplementary Information).

### Experimental procedures

#### Total RNA purification and quantitation

Total RNA was purified from 1 mL human whole blood (stabilised with RNA*later*™ and stored at −80 °C) using the Ambion™ RiboPure™ RNA Purification Kit, blood (Thermo Fisher Scientific, Norwood SA, Australia). Purified total RNA was then immediately quantitated using a Qubit® 2.0 Fluorometer before storage at −80 °C in 1 μg aliquots ready for cDNA library preparation. An additional 10 μL aliquot was also made in order to enable quantification of RNA integrity number (RIN) using the Agilent RNA 6000 Nano Kit and an Agilent 2100 Bioanalyzer.

#### cDNA library preparation and RNA sequencing

cDNA libraries were prepared with 1 μg total RNA template using the TruSeq Stranded Total RNA with Ribo-Zero Globin kit (Illumina, Scoresby VIC, Australia). Libraries were sequenced using the Illumina HiSeq 4000 in 75 bp paired-end read mode.

### Bioinformatics procedures and analyses

#### Processing of RNA sequencing data

Basic quality checks were run using FastQC (Andrews: http://www.bioinformatics.babraham.ac.uk/projects/fastqc/) followed by adapter and quality (-q option set to 20) trimming with Cutadapt (v1.12) in paired-end mode^40^. Remaining reads were pseudoaligned to the human transcriptome (as per GRCh38.84 GTF file downloaded from Ensembl on 08/03/2016) and transcript abundances quantified using Kallisto (v0.44.0)^41^. The tximport R package (v1.8.0)^42^ was then used to summarise transcript abundances to the gene level.

#### Gene co-expression network analysis

Gene features with read counts of less than 10 in greater than 90% of samples were first removed and the remaining data were regularised log (rlog) transformed using the DESeq2 R package^43^. Residuals after adjusting for age, sex, and RIN were then used as input for weighted gene co-expression network analysis (WGCNA) using the WGCNA R package (1.62)^34^ (Supplementary Information).

#### Enrichment and pathway analyses

Functional term and pathway enrichment analyses were performed using the functional annotation clustering tool implemented within the recently updated Database for Annotation, Visualisation, and Integrated Discovery (DAVID) (v6.8)^44^ (Supplementary Information). For additional exploration of gene networks and canonical pathways associated with lithium response, we used QIAGEN’s Ingenuity® Pathway Analysis (IPA®, QIAGEN Redwood City, www.qiagen.com/ingenuity).

## Results

### Network construction and prioritisation of a lithium response-associated module

We constructed a weighted gene co-expression network using regularised log transformed count data for 13,659 genes from 50 lithium-treated BPAD cases (see methods for details of filtering). The resulting network comprised 23 modules ranging in size between 31 and 2912 genes (Supplementary Fig. 1).

**Fig. 1:**
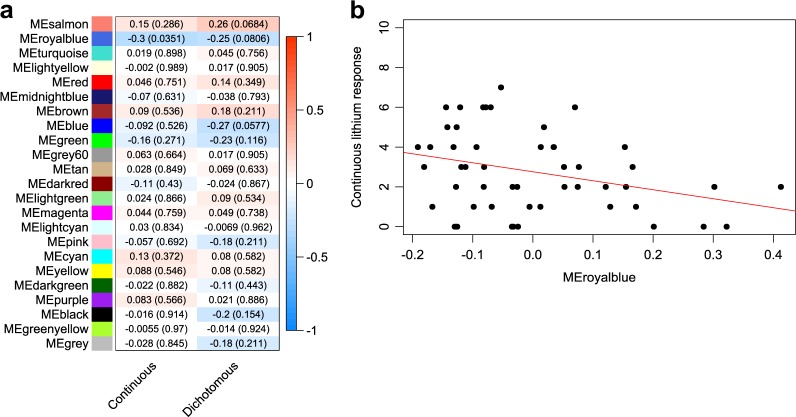
Identification of a co-expression module relevant for lithium response in BPAD. **a** Heatmap depicting correlations (*p*-values) between 23 co-expression module eigengenes (MEs) and lithium response represented as a continuous and a dichotomous phenotype. **b** Scatterplot with a line of best fit illustrating the relationship between the royalblue ME and lithium response represented as a continuous phenotype. Colours correspond to different modules from the overall co-expression network. ME module eigengene

To identify any potential lithium response-relevant modules within our network, we first computed eigengene composite values (Supplementary Information)^35^ for each module within each of our participants and correlated (Spearman’s rank) them with lithium treatment response scores expressed in both a continuous and dichotomous fashion (see methods). Overall, we found one nominally significant correlation between continuous lithium response and the eigengene of a module denoted ‘royalblue’ (cor = −0.299, *p* = 0.035) (Fig. 1a). The correlation between this module and dichotomous lithium response did not meet the nominal significance threshold, though the trend was similar (cor = −0.249, *p* = 0.081) (Fig. 1a). The negative sign on these correlations signifies that the overall expression profile of this module was lower in those participants with higher lithium response scores relative to those with lower scores (Fig. 1a, b).

In addition, the royalblue module was also significantly correlated with total B scores from the ALDA scale (Supplementary Information) (cor = 0.322, *p* = 0.023) (Supplementary Fig. 2a, b). Notably, there were no additional nominally significant (*p* < 0.05) correlations between the royalblue module and any items from a panel of assessed psychiatric features (Supplementary Fig. 2d).

### Characterisation of a lithium response-associated module

**Fig. 2:**
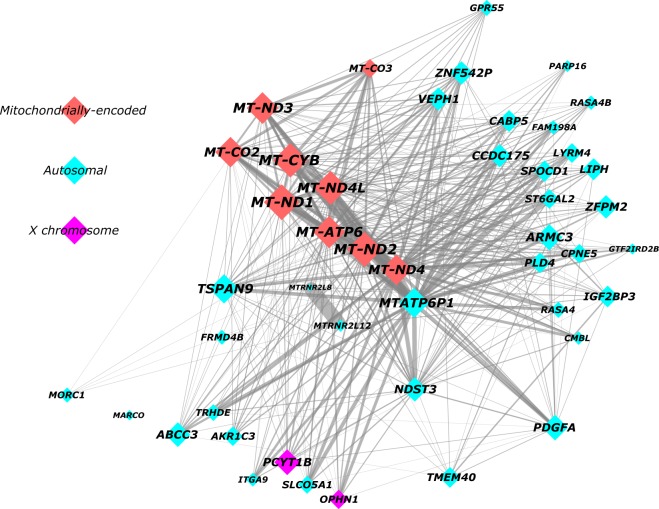
Network graph summarising the royalblue co-expression module. The royalblue module was visualised according to a prefuse force directed layout based on weighted correlations between genes. Minor manual changes to node placement were made to maximise clarity. The size of each node (and node label) reflects absolute module membership (MM) values, with larger nodes corresponding to higher MM values. Node colour indicates whether royalblue genes were encoded by autosomal (azure), X chromosome (purple), or mitochondrial (red) DNA. Edge width reflects the weighting of connections between nodes, with thicker edges corresponding to stronger connections

The royalblue module consists of 46 genes in total (Fig. 2; Supplementary Table 2); 44 of which are protein-coding while the remaining two encode pseudogenes. A look at the chromosomal distribution of royalblue module genes revealed that 35 are autosomal, 2 reside on the X chromosome, whereas the remaining 9 are all mitochondrially-encoded (Fig. 2).

#### Module membership and gene significance

In order to identify the central regulators (or hub genes) of the royalblue module, we computed the corresponding module membership (MM) values (see methods). The gene with the highest absolute MM was *MT-ND1* (MM = 0.939, *p* = 7.05E-24) (Table 1a), which encodes the Mitochondrially-Encoded NADH: Ubiquinone Oxidoreductase Core Subunit 1 protein. Further, when we pulled out all genes with a MM > abs(0.8) for inspection (Table 1a), which yielded the top ten genes, the first eight of them were all mitochondrially-encoded, the ninth was an autosomal mitochondrial pseudogene (*MTATP6P1*), whereas the tenth was an autosomal protein-coding gene called *TSPAN9*. Thus, the mitochondrially-encoded genes within the royalblue module are clearly the central regulators (Table 1a; Fig. 2).

**Table 1a:** Top ten genes from the royalblue module with the highest module membership values

Ensembl ID	HGNC symbol	Chr	Description	MM	*p*	GS	*p*
ENSG00000198888	*MT-ND1*	MT	Mitochondrially-encoded NADH:ubiquinone oxidoreductase core subunit 1	0.939	7.05^E-24^	−0.195	0.174
ENSG00000198763	*MT-ND2*	MT	Mitochondrially-encoded NADH:ubiquinone oxidoreductase core subunit 2	0.929	2.04^E-22^	−0.209	0.145
ENSG00000198727	*MT-CYB*	MT	Mitochondrially-encoded cytochrome b	0.922	2.05^E-21^	−0.273	0.055
ENSG00000212907	*MT-ND4L*	MT	Mitochondrially-encoded NADH:ubiquinone oxidoreductase core subunit 4L	0.902	3.87^E-19^	–0.244	0.087
ENSG00000198840	*MT-ND3*	MT	Mitochondrially-encoded NADH:ubiquinone oxidoreductase core subunit 3	0.890	5.40^E-18^	–0.264	0.064
ENSG00000198899	*MT-ATP6*	MT	Mitochondrially-encoded ATP synthase 6	0.877	6.98^E-17^	–0.322	0.023
ENSG00000198712	*MT-CO2*	MT	Mitochondrially-encoded cytochrome c oxidase II	0.827	1.44^E-13^	–0.229	0.110
ENSG00000198886	*MT-ND4*	MT	Mitochondrially-encoded NADH:ubiquinone oxidoreductase core subunit 4	0.823	2.19^E-13^	–0.231	0.106
ENSG00000248527	*MTATP6P1*	1	Mitochondrially-encoded ATP synthase 6 pseudogene 1	0.807	1.43^E-12^	–0.157	0.276
ENSG00000011105	*TSPAN9*	12	Tetraspanin 9	0.803	2.31^E-12^	–0.275	0.053

MM module membership to the royalblue module, *GS* gene significance for lithium response represented as a continuous phenotype

We also computed gene significance (GS) values to determine which individual genes within the royalblue module were most highly correlated with continuous lithium response. The gene with the highest absolute GS was *CPNE5*, which is an autosomal gene encoding a calcium-dependent lipid-binding protein called Copine 5 (GS = −0.416, *p* = 0.003) (Table 1b). Of the ten genes with the highest GS, two of them were mitochondrially-encoded indicating that the apparent mitochondrial-related functioning of the royalblue module is also relevant for its association with lithium response (Table 1b). Accordingly, we observed a small correlation between absolute MM and absolute GS scores for continuous lithium response across the royalblue module, though this did not meet the nominal significance threshold (cor = 0.18, *p* = 0.23) (Supplementary Fig. 3). Notably, for all but 3 of the 46 royalblue genes (*MARCO*, *AKR1C3*, *PLD4*) the GS values were negatively signed (Supplementary Table 2**)**, which again indicates that the overall expression profile of the royalblue module was downregulated in participants with better lithium response.

**Table 1b:** Top ten genes from the royalblue module with the highest gene significance values

**Ensembl ID**	HGNC symbol	Chr	Description	MM	*p*	GS	*p*
ENSG00000124772	*CPNE5*	6	Copine 5	0.528	8.22^E-05^	–0.416	0.003
ENSG00000102230	*PCYT1B*	X	Phosphate cytidylyltransferase 1, choline, beta	0.777	3.16^E-11^	–0.341	0.015
ENSG00000105808	*RASA4*	7	RAS p21 protein activator 4	0.364	0.009	–0.325	0.021
ENSG00000165309	*ARMC3*	10	Armadillo repeat containing 3	0.695	2.22^E-08^	–0.324	0.022
ENSG00000198899	*MT-ATP6*	MT	Mitochondrially-encoded ATP synthase 6	0.877	6.98^E-17^	–0.322	0.023
ENSG00000144668	*ITGA9*	3	Integrin subunit alpha 9	0.415	0.003	–0.304	0.032
ENSG00000137571	*SLCO5A1*	8	Solute carrier organic anion transporter family member 5A1	0.550	3.45^E-05^	–0.285	0.045
ENSG00000136231	*IGF2BP3*	7	Insulin like growth factor 2 mRNA binding protein 3	0.605	3.26^E-06^	–0.282	0.047
ENSG00000011105	*TSPAN9*	12	Tetraspanin 9	0.803	2.31^E-12^	–0.275	0.053
ENSG00000198727	*MT-CYB*	MT	Mitochondrially-encoded cytochrome b	0.922	2.05^E-21^	–0.273	0.055

*MM* module membership to the royalblue module, *GS* gene significance for lithium response represented as a continuous phenotype

#### Term and pathway enrichment analyses

**Fig. 3:**
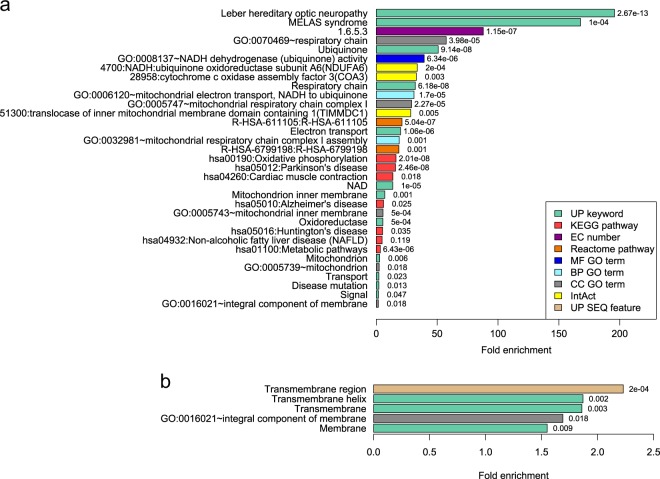
Enrichment analyses of royalblue module genes. Bar charts summarising two significant functional annotation clusters indicating enrichment of **a** mitochondrial-related genes and **b** genes encoding membrane proteins. Fold enrichment of individual terms within each cluster is indicated by bar length, and corresponding uncorrected *p*-values are indicated to the right of each bar. Note the difference in fold enrichment scale between **a** and **b** panels. UP uniProt, KEGG kyoto encyclopaedia of genes and genomes, EC enzyme commission, GO gene ontology, MF molecular function, BP biological process, CC cellular component, IntAct molecular interaction database, SEQ sequence annotation

In order to functionally characterise the royalblue module we performed term and pathway enrichment analyses using the functional annotation clustering tool available at the Database for Annotation, Visualisation and Integrated Discovery (DAVID) (Supplementary Information)^44^. Overall, there were two significant functional annotation clusters with an enrichment score > 1.3 (which corresponds to *p* < 0.05) (Fig. 3; Supplementary Table 3).

The top cluster, which had an enrichment score of 4.1, consisted of 33 annotation terms and was driven primarily by the mitochondrially-encoded genes within the module. Among the top annotation terms within this cluster were multiple mitochondrial-related Uniprot keywords including ‘Respiratory chain’ (32-fold enrichment), ‘Ubiquinone’ (51-fold), and ‘Electron transport’ (20-fold); while the top functional pathways include ‘Oxidative phosphorylation’ (16-fold) (KEGG; hsa00190) and ‘Respiratory electron transport’ (21-fold) (Reactome; R-HSA-611105) (Fig. 3a; Supplementary Table 3). Of particular note, there was also a gene ontology (GO) cellular component (CC) term specifically highlighting complex I of the respiratory chain (29-fold), which was associated with five paralogous royalblue mitochondrially-encoded genes (*MT-ND1*, *2*, *3*, *4*, and *4L*).

The second functional annotation cluster had an enrichment score of 2.6 and was composed of five annotation terms in total, all of which indicate a general overrepresentation of royalblue-encoded proteins localised to membranes. The fold-enrichments here were much more subtle, with a range between 1.5- and 2.2-fold. Notably, the mitochondrially-encoded genes did contribute towards these enrichments though they were not the main drivers (Fig. 3b; Supplementary Table 3).

**Fig. 4:**
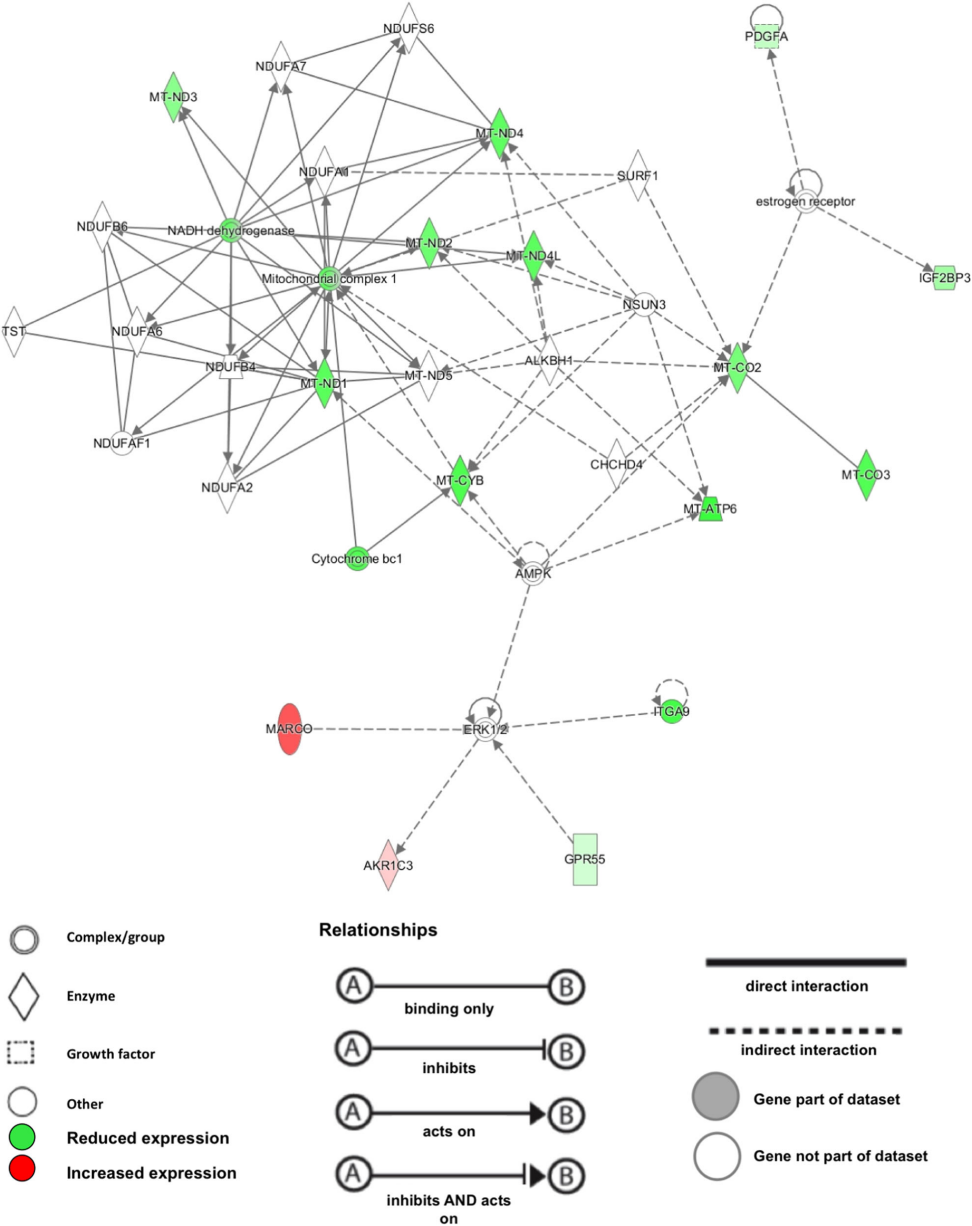
Graphic representation of the IPA® top gene network derived from the royalblue module. Green colour indicates reduced gene expression in better lithium treatment responders, relative to poor responders. Red colour indicates increased gene expression, respectively. The mitochondrial complex 1 is identified as the central functional hub within the royalblue module

To further explore and validate these functional findings, we subjected the royalblue module to Ingenuity® Pathway Analysis (IPA®). Canonical pathway analysis confirmed the centrality of mitochondrial mechanisms to the module, and listed ‘oxidative phosphorylation’ (*p* = 7.16E-13, 8.3% pathway overlap) and ‘mitochondrial dysfunction’ (4.28E-11, 5.3% pathway overlap) as top hits (supplementary Table 4). IPA® analysis of functional networks revealed as top hit a network with the annotations ‘metabolic disease, developmental disorder, hereditary disorder’. This network achieved an IPA® network score of 35, indicating that the overlap between this network and the royalblue module genes is highly significant (i.e., an IPA® network score of > 2 corresponds to *p* < 0.01). Visualisation of this network identified the downregulation of mitochondrial complex 1 as the central functional hub within the royalblue module (Fig. 4).

## Discussion

To identify biological systems and mechanisms associated with lithium response in BPAD, we performed co-expression network analyses of whole-blood-derived RNA sequence data from *n* = 50 BPAD cases with lithium response data. We identified a module (denoted ‘royalblue’) comprising 46 genes that was negatively correlated, at nominal significance, with lithium response represented as a continuous phenotype. Functional characterisation of this module by term enrichment and pathway analyses revealed that its central regulators (9 out of 46 genes) are mitochondrially-derived, and encode components of the electron transport chain (ETC) and oxidative phosphorylation (OXPHOS) pathway. These mechanisms fulfil multiple roles, chief among them being the production of adenosine triphosphate (ATP), the cell’s primary source of chemical energy^45,46^.

Reassuringly, the findings from our study are in keeping with previous literature relating to BPAD and lithium response. Firstly, numerous gene expression studies in both peripheral and central tissues have already shown altered mRNA levels of multiple mitochondrial-related genes in BPAD patients relative to controls^47–49^. This includes a number of the mitochondrially-encoded genes found to be altered in the present study such as *MT-ND2* and *MT-ATP6* among others. Secondly, in addition to evidence from gene expression studies, there are many other lines of evidence ranging from post-mortem, genetic, brain imaging, peripheral cell, and animal studies that point to general mitochondrial dysfunction in the pathophysiology of BPAD^50–52^. And thirdly, lithium treatment has been shown on multiple occasions to impact mitochondrial functioning, at least in part by modulating ETC and OXPHOS pathway activity^53,54^.

Of particular note is a recent landmark study that generated dentate gyrus (DG) granule cell-like neurons using induced pluripotent stem cells (iPSCs) collected from six BPAD patients and four healthy controls^55^. RNA-seq analyses of these cells highlighted 45 genes as being significantly (FDR < 0.1) differentially expressed between patients and controls, which included six mitochondrially-encoded transfer RNAs that were all enhanced in BPAD neurons. In accordance with this, the authors observed enhanced mitochondrial membrane potential (MMP) in BPAD DG-like neurons, which is indicative of increased mitochondrial functioning.

Crucially, the BPAD patients from this study were selected based on their known response to lithium treatment; three ‘lithium responders’ (LR) and three ‘non-responders’ (NR)^55^. Following chronic lithium treatment, RNA-seq analyses revealed 560 differentially expressed genes in LR DG-like neurons relative to control, compared to just 40 genes from the NR group. Further, 84 of these differentially expressed genes from the LR group were found to have been rescued by lithium treatment, including ten mitochondrially-encoded genes that were downregulated in response to chronic lithium. Strikingly, 7 of these 10 mitochondrial genes belong to the royalblue module identified in the present study, all of which we found had lower mRNA expression in better lithium responders relative to poorer.

Thus, the directionality observed in whole blood in our study with regards to mitochondrially-encoded gene expression is consistent with that previously seen in DG-like neurons derived from iPSCs^55^. This consistency offers convincing support for the validity and robustness of our findings, which is underscored not only by the obvious difference between the two studies in terms of tissue, but also the difference in phenotype definition. Indeed, in our study we utilised the ALDA scale to define lithium response both as a continuous and a dichotomous variable, where we observed a nominally significant association with the continuous phenotype only. In contrast, the iPSC study used the Clinical Global Impressions Scale to define a dichotomous phenotype; i.e., LR vs. NR^55^. However, it is important to note that only two BPAD patients in our study achieved a total ALDA score ≥ 7, which according to ConLi^+^Gen is the optimum cutoff to define a dichotomous phenotype^39^. We, therefore, used a lower cutoff of 5 (Supplementary Information), which may explain why we did not observe a significant correlation with our dichotomous phenotype.

Nevertheless, given there is ample precedence for a relationship between mitochondrial functioning, BPAD, and lithium response, one of the major questions going forward is to determine the precise mechanisms underlying this relationship. One intriguing line of investigation points to a possible mediating role for neuronal excitability. Previous studies have reported hyperexcitable neurons within the VTA and DG of animal models of BPAD, which is considered to be a potential endophenotype of BPAD in humans^56,57^. In the aforementioned iPSC study, patch-clamp recordings revealed that iPSC-derived DG-like neurons from BPAD patients are indeed hyperexcitable relative to healthy control neurons^55^. Furthermore, they also showed that this phenotype could be selectively rescued by chronic lithium treatment; though intriguingly this effect was only observed in the LR neurons, not the NR neurons. Given the authors observed this exact same pattern with regards to mitochondrially-encoded mRNA expression^55^, this suggests mitochondrial functioning and neuronal excitability in DG-like neurons could be intimately linked, and may act synergistically to modulate lithium response in BPAD.

Although this proposed link is circumstantial at present, it does make intuitive sense given that neuronal excitability is inherently dependent on ATP produced by the ETC and OXPHOS pathways. It is noteworthy that in a follow-up study using an independent sample of iPSCs, the authors observed subtle differences in electrophysiological measures between LR and NR DG-like neurons^58^, indicating that the mechanisms driving neuronal hyperexcitability in these two groups of BPAD patients may be distinct. Strikingly, the authors were able to use these electrophysiological differences in DG-like neurons to predict the responsiveness of a new patient to lithium with a success rate of over 92%. Thus, if the proposed links between hyperexcitability, mitochondrial functioning, and lithium responsiveness support the notion of a discrete lithium-responsive phenotype, this inspires genuine hope for the future of personalised medicine in BPAD, particularly if the underlying mitochondrial dysregulation can be detected in the periphery as findings from the present study suggest.

Nevertheless, as a first priority the correlation observed in the present study between the royalblue module and lithium response requires replication, preferably in a larger independent sample of BPAD patients. To the best of our knowledge a genuine ‘like-for-like’ independent replication sample with whole-blood transcriptomic data is not currently available, and so we urge future studies to address this gap. The closest replication sample we were able to find, albeit with a smaller sample size, comprised of RNA-seq data from cultured lymphoblastoid cell lines (LCLs) derived from 24 BPAD patients; 12 LRs (total ALDA ≥ 7) and 12 NRs (total ALDA < 7). We note that we were unable to replicate our findings in this independent sample using either a dichotomous or a continuous phenotype definition (data not shown). However, the core mitochondrial genes of the royalblue module did not survive the filtering process in this data set, rendering replication unlikely, and so given the clear differences between this sample (in vitro) and ours (ex vivo), we believe further replication attempts in more comparable samples are necessary.

We also urge comprehensive functional validation in future independent replication samples to determine the downstream effects of royalblue module dysregulation at the mRNA level. For example, is lithium response significantly correlated with the royalblue module at the protein level as measured in serum or plasma? Even further downstream than this, we suggest metabolic measures to capture ETC and OXPHOS activity, along with MMP and mitochondrial morphology, should feature on a panel of mitochondrial phenotypes for comparison between better and poorer lithium responders. As already alluded to, findings from these types of assays could prove to be vital for informing the development of mitochondrial biomarkers to help predict lithium response in BPAD.

A major limitation of the present study relates to the cause-effect conundrum that plagues gene expression studies in general. This issue is further compounded in our case by the retrospective nature of our lithium treatment response measure. For example, the measure cannot ultimately distinguish lithium effects from mechanisms underlying spontaneous remission from BPAD episodes, which have been described extensively in the historic literature^59^. It is not possible to disentangle this issue based on these data alone, though genetic association data can provide useful information. Although mitochondrial DNA variation has so far received little attention in relation to lithium response in BPAD, there has been a report of a significant association between the mtDNA 10398A polymorphism and lithium response^60^. This polymorphism results in an amino acid substitution in *MT-ND3*, which is one of the mitochondrially-encoded genes comprising the royalblue module. Thus, future work is required to determine whether the regulation of the royalblue module may in fact be driven by mtDNA polymorphism.

To conclude, in the present study we highlight a peripheral blood co-expression module comprising 46 genes that may be of relevance for lithium treatment response in BPAD patients. This module is heavily enriched with mitochondrial-related genes involved primarily in the ETC and OXPHOS, and the expression of these genes was found to be lower in better lithium responders relative to poorer. Importantly, our findings are pre-validated by previous transcriptomic studies supportive of an association not only between mitochondrially-encoded mRNA expression levels and BPAD, but also lithium response in BPAD. Furthermore, the apparent dysregulation of the royalblue module observed here is subtle, which speaks to the sensitivity of co-expression network analysis approaches while underscoring the importance of complementing traditional SNP- and gene-centric approaches with systems biology methodology.

## Electronic supplementary material

Suppl fig 3

Suppl fig 2

Suppl fig 1

Suppl information

suppl tables

Supplemental Legends
