# Supplementary figures and images for: A gene co-expression module implicating the mitochondrial electron transport chain is associated with long-term response to lithium treatment in bipolar affective disorder
Source: Transl Psychiatry. 2018 Sep 5;8:183. doi: 10.1038/s41398-018-0237-0 (PMC6125294; doi:10.1038/s41398-018-0237-0)

## Supplementary Figure 3

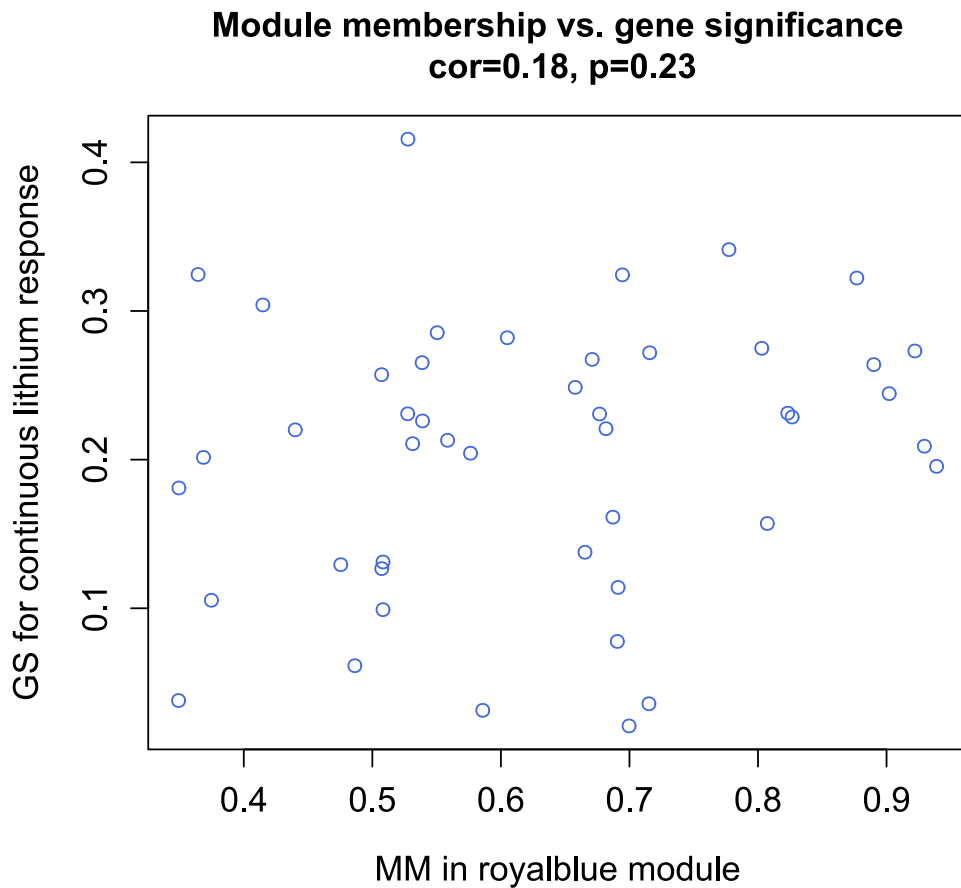

Supplement: Supplementary file 1 — Suppl fig 3 [file 41398_2018_237_MOESM1_ESM.pdf]

## Supplementary Figure 2

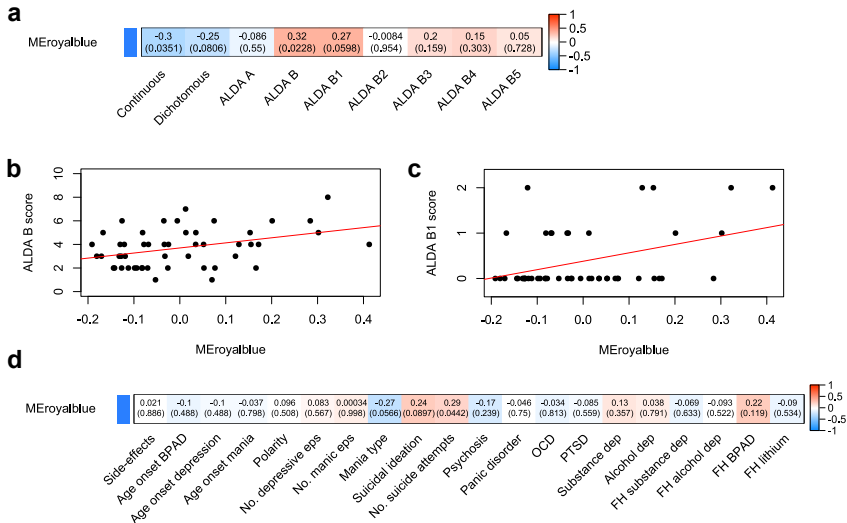

Supplement: Supplementary file 2 — Suppl fig 2 [file 41398_2018_237_MOESM2_ESM.pdf]

# Supplementary Figure 1

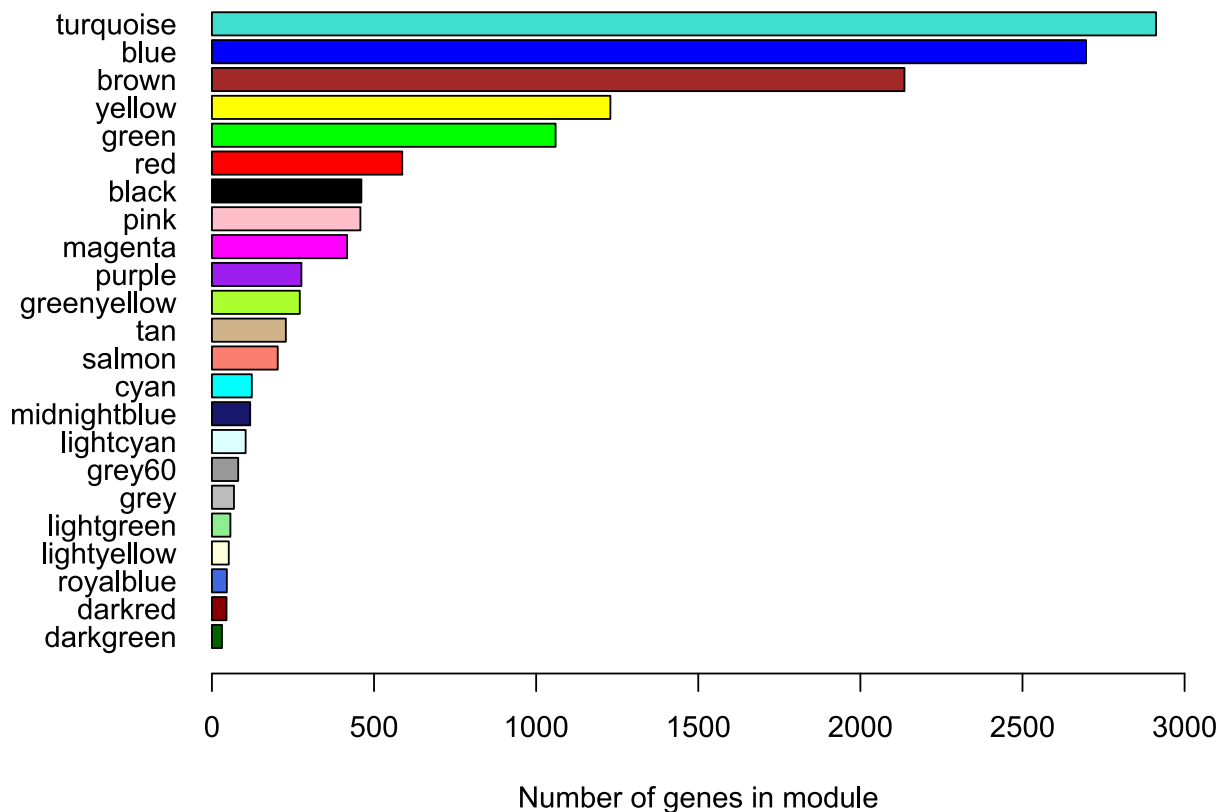

Supplement: Supplementary file 3 — Suppl fig 1 [file 41398_2018_237_MOESM3_ESM.pdf]
