# Supplementary material for: A gene co-expression module implicating the mitochondrial electron transport chain is associated with long-term response to lithium treatment in bipolar affective disorder
Source: Transl Psychiatry. 2018 Sep 5;8:183. doi: 10.1038/s41398-018-0237-0 (PMC6125294; doi:10.1038/s41398-018-0237-0)
Supplement: Supplementary file 4 — Suppl information [file 41398_2018_237_MOESM4_ESM.docx]

**Supplementary information**

**The International Consortium on Lithium Genetics**

The International consortium on Lithium Genetics (ConLi^+^Gen) was ConLi^+^Gen was formed to bring together samples of lithium-treated BPAD patients from around the world with standardized clinical assessment, including general demographics, detailed information on the course of participants’ bipolar illness, psychiatric and physical comorbidities, medication and substance history, and family psychiatric history ^1^.

**The ALDA scale**

In the present study lithium treatment response was measured using the ALDA scale, which provides a retrospective, self-report, measure of long-term treatment response in research participants with bipolar disorder ^2, 3^. Briefly, the scale is composed of an A criterion and multiple B criteria. For the A criterion a score between 0 and 10 is assigned depending on the extent of clinical improvement; i.e., a score of 0 represents no change (or worsening) in symptoms and a score of 10 corresponds to a complete response. On the other hand, the B criteria are designed to determine the extent to which there was a causal relationship between the treatment and clinical improvement. There are five B criteria in total, each scored between 0 and 2, measuring the following: (B1) number of episodes before/off treatment, (B2) frequency of episodes before treatment, (B3) duration of the treatment, (B4) compliance during period(s) of stability, and (B5) the use of additional medication during the period(s) of stability. A total B score is then calculated by summing the scores from the five B criteria, after which the total ALDA score is calculated by subtracting the total B score from the A score. For participants with a total B score greater than their A score, the total ALDA score is set to 0.

In the present study we directly utilised the total ALDA score (i.e., range 0-10) as a “continuous” measure of lithium treatment response. Furthermore, we also defined a “dichotomous” variable. In a multistage inter-rater reliability study conducted by ConLi^+^Gen, the authors found that the most reliable dichotomous phenotype was one that defined those with a total ALDA score ≥7 as “responders” ^1^. However, total ALDA score cut-offs of ≥6 and ≥5 were also found to be highly reliable. In our sample, we had just a single participant with a total ALDA score ≥7, seven with ≥6, and ten with ≥5, and so we therefore chose a score of ≥5 as our dichotomous cut-off to define “non-responders” vs. “responders”.

**Weighted gene co-expression network analysis (WGCNA)**

Briefly, Pairwise Pearson’s correlations were computed for all gene features across all samples, and a power function parameter of β=6 (i.e., smallest β that led to an approximately scale-free network with the truncated scale-free fitting index R^2^>0.8 in our data) was applied to generate an adjacency matrix. Modules were detected using a basic tree cut algorithm and module eigengenes defined as the first principal component of the standardised gene expression profile ^4^ were computed. Module eigengenes (MEs) were then correlated with continuous and dichotomous (see above) measures of lithium response, as well as various other measured psychiatry-related features. Because all of the above phenotypes were either dichotomous/ordinal or were not normally distributed, we used the Spearman’s rank correlation coefficient for all ME-phenotype correlations. Likewise, for the same reasons we also computed module membership (MM) and gene significance (GS) values using the Spearman’s rank correlation coefficient.

**Functional annotation clustering**

Briefly, the functional annotation clustering tool available at DAVID returns clusters of functionally-related terms and pathways with uncorrected and corrected *p*-values for each individual term/pathway within each cluster, as well as an overall enrichment score for each cluster. An enrichment score >1.3 is considered equivalent to significance at a nominal level (i.e., *p*<0.05) ^5^. The background gene set consisted of all genes inputted into WGCNA after filtering (a total of 13,659 genes).

**References**

1. Manchia M, Adli M, Akula N, Ardau R, Aubry JM, Backlund L *et al.* Assessment of Response to Lithium Maintenance Treatment in Bipolar Disorder: A Consortium on Lithium Genetics (ConLiGen) Report. *PloS one* 2013; **8**(6)**:** e65636.

2. Duffy A, Alda M, Milin R, Grof P. A consecutive series of treated affected offspring of parents with bipolar disorder: is response associated with the clinical profile? *Can J Psychiatry* 2007; **52**(6)**:** 369-376.

3. Garnham J, Munro A, Slaney C, Macdougall M, Passmore M, Duffy A *et al.* Prophylactic treatment response in bipolar disorder: results of a naturalistic observation study. *J Affect Disord* 2007; **104**(1-3)**:** 185-190.

4. Langfelder P, Horvath S. Eigengene networks for studying the relationships between co-expression modules. *BMC Syst Biol* 2007; **1:** 54.

5. Huang da W, Sherman BT, Lempicki RA. Systematic and integrative analysis of large gene lists using DAVID bioinformatics resources. *Nature protocols* 2009; **4**(1)**:** 44-57.
