# Supplementary material for: A gene co-expression module implicating the mitochondrial electron transport chain is associated with long-term response to lithium treatment in bipolar affective disorder
Source: Transl Psychiatry. 2018 Sep 5;8:183. doi: 10.1038/s41398-018-0237-0 (PMC6125294; doi:10.1038/s41398-018-0237-0)
Supplement: Supplementary file 6 — Supplemental Legends [file 41398_2018_237_MOESM6_ESM.docx]

**Supplementary Legends**

**Supplementary Figure 1. Bar chart depicting the number of genes comprising each module of our co-expression network**

**Supplementary Figure 2. Correlations between the royalblue module and individual ALDA scale components as well as various psychiatric-related features.**

(**a**) Heatmap depicting correlations (*p*-values) between the royalblue ME and individual components of the ALDA scale. (**b** and **c**) Scatterplots with lines of best fit illustrating the relationship between the royalblue ME and the total B score (**b**) as well as the B1 score (**c**) components of the ALDA scale. (**d**) Heatmap depicting the correlations (*p*-values) between the royalblue ME and multiple BPAD, lithium, and other psychiatric-related features of our participants. ME; module eigengene, BPAD; bipolar affective disorder, OCD; obsessive-compulsive disorder, PTSD; post-traumatic stress disorder, FH; family history

**Supplementary Figure 3. Scatter plot depicting the correlation between module membership (MM) and gene significance (GS) values**
